# Bamboo leaf flavone changed the community of cecum microbiota and improved the immune function in broilers

**DOI:** 10.1038/s41598-020-69010-1

**Published:** 2020-07-23

**Authors:** Gang Shu, Fanli Kong, Dan Xu, Lizi Yin, Changliang He, Juchun Lin, Hualin Fu, Kaiyu Wang, Yaofu Tian, Xiaoling Zhao

**Affiliations:** 1 Department of Basic Veterinary Medicine, Sichuan Agricultural University, Chengdu, 611130 Sichuan China; 2 College of Life Science, Sichuan Agricultural University, Ya’an, 625014 Sichuan China; 3 Farm Animal Genetic Resources Exploration and Innovation Key Laboratory of Sichuan Province, Sichuan Agricultural University, Chengdu, 611130 Sichuan China

**Keywords:** Pharmacology, Immunology, Microbiology

## Abstract

It has been shown that bamboo leaf flavone (BLF) displays biological and pharmacological activities in mammals. However, the effects of BLF on broiler gut microbiota and related immune function have not been investigated. The aim of this study was to test our hypothesis that BLF can improve the health status of broilers by modulating the gut microbiota. A total of 300 one-day-old Arbor Acres (AA) broilers were used to characterize their gut microbiota and immune status after feeding diet supplemented with BLF. The V4 hypervariable region of the 16S rRNA gene from cecal bacteria was sequenced via the Illumina MiSeq platform. The Immune status and related parameters were assessed, including the immune organ index (the spleen, thymus, and bursa), serum concentrations of IL-2 and INF-γ, and spleen *IL-2* and *INF-γ* gene expressions. The results showed the BLF diet had an Immune enhancement effect on broilers. In addition, BFL caused the changes of the gut microbial community structure, resulting in greater proportions of bacterial taxa belonging to *Lactobacillus*, *Clostridiales*, *Ruminococcus*, and *Lachnospiraceae*. These bacteria have been used as probiotics for producing short chain fatty acids in hosts. These results indicate that BLF supplement improves immune function in chicken via modulation of the gut microbiota.

## Introduction

Flavonoids are polyphenolic compounds that ubiquitously present in plants. Flavonoid-rich foods and flavonoid supplements have been found to modulate immune function by inducing cytoprotective effects^1^, restoring lymphocyte proliferation^2^, and preventing cells apoptosis in mammals^3^. The flavonoid extracted from bamboo leaves (so-called bamboo leaf flavone, BLF), mainly including orientin, homoorientin, vitexin, and isovitexin^4^, have been confirmed to have multiple biological activities, such as scavenging oxygen radicals^5^, enhancing immunity^6^, and possessing anticancer^7^, antibacterial^8^, antiviral^9^, and antioxidant functions^10^. So, it has been widely used as drug substances, anti-aging products, cosmetics, and feed stuffs in human. However, its uses and roles in livestock production are limited^11^. The ban and restrictions on the use of antibiotics in livestock production have directed researches to investigate natural or organic alternatives to antibiotics in plants (e.g. BLF) with the potential roles to promote growth and health as well as production performance such as meat, milk, egg production^11^.

The microbiota communities in the gut play significant roles in promoting health status and productivity in chickens^12^ by modulating their immune system, inhibiting pathogenic colonization, and promoting detoxification and digestion^13^. Recent studies have reported that gut microbiota involve in transforming flavonoid into phenolic acids, which can be easily absorbed in mice^14^. Huang et al.^15^ reported that flavonoids extracted from plants including quercetin, catechin, and puerarin had impact on the relative viability of the gut microbiota, which implies that dietary flavonoids supplements play an important role in reshaping the gut microbial community, providing beneficial effects such as Immunoenhancement in hosts. To our knowledge, few reports on the use of BLF to regulate the gut microbiota and immune function in animals have been investigated.

Therefore, the aim of this study was to investigate effect of BLF on broiler cecal microbiota and related immunity index during various developing stages that are day 14 (starter stage), 28 (grower stage), and 42 (finisher stage). The results indicated that BFL is applicable as a natural feed additive for chickens to boost their health and production.

## Results

### Immune organs index, serum concentrations of IL-2 and INF-γ and their gene expression abundances in spleen

The positive effects of BFL on broiler vital immune organs (the thymus, spleen, and bursa of fabricius) are presented in Table 1. Compared to controls, spleen index was increased in all BLF fed broilers at day 42 although the significant increase was found in the M group only (*P* < 0.05). Similarly, the index of bursa of fabricius was increased in both M and H groups at day 28 (*P* < 0.05). The thymus index was increased in both L and H groups at day 14 (*P* < 0.05), and continuously increased in H group at day 24 (*P* < 0.05).

**Table 1:** Effect of bamboo leaf flavone supplementation on broiler immune organs index.

Organs	Growth point (day)	Treatments			
		Control	L	M	H
Spleen	14	0.93 ± 0.14	0.97 ± 0.15	1.00 ± 0.07	1.07 ± 0.20
	28	0.82 ± 0.06	0.91 ± 0.15	1.06 ± 0.22	1.05 ± 0.17
	42	1.09 ± 0.13^b^	1.31 ± 0.20^ab^	1.35 ± 0.06^a^	1.22 ± 0.15^ab^
Thymus	14	2.48 ± 0.48^b^	3.64 ± 1.96^a^	3.10 ± 1.23^ab^	3.98 ± 0.88^a^
	28	3.56 ± 0.49^b^	5.20 ± 1.89^ab^	5.35 ± 1.88^ab^	5.63 ± 0.99^a^
	42	5.53 ± 0.92	5.61 ± 0.63	5.90 ± 1.26	5.73 ± 0.99
Bursa of fabricius	14	2.19 ± 0.61	2.02 ± 0.33	2.42 ± 0.27	2.38 ± 0.22
	28	2.25 ± 0.36^c^	2.36 ± 0.53^bc^	3.23 ± 0.28^b^	3.93 ± 0.54^a^
	42	2.55 ± 0.20	2.71 ± 0.27	3.09 ± 0.49	2.88 ± 0.45

Data were presented as mean ± SD.

^a–^^c^Values in the same row without the same uppercase differed significantly (*P* < 0.05).

Bamboo leaf flavone effects on serum IL-2 and INF-γ concentrations in broilers were summarized in Table 2. Compared to controls, IL-2 concentrations were significantly increased in both M and H groups at day 28 (*P* < 0.05), which were continuously increased in all treatment groups on day 42 (*P* < 0.05). Moreover, compared to control group, the concentrations of IFN-γ were increased in all the experimental groups at day 28 (*P* < 0.05); while at day 42, the upregulation was seen in M group only (*P* < 0.05).

**Table 2:** Treatment effects on the serum concentrations of IL-2 and IFN-γ.

Immune factors (ng/L)	Growth point (day)	Treatments			
		Control	L	M	H
IL-2	14	42.13 ± 2.32	44.2 ± 3.15	45.5 ± 2.11	44.4 ± 2.12
	28	45.43 ± 3.13^b^	46.5 ± 4.21^b^	55.4 ± 4.14^a^	58.7 ± 3.11^a^
	42	26.12 ± 3.14^b^	32.2 ± 4.28^a^	34.2 ± 1.50^a^	31.1 ± 2.10^a^
IFN-γ	14	11.21 ± 0.88	11.56 ± 0.56	12.01 ± 0.72	11.44 ± 0.91
	28	8.21 ± 0.65^b^	9.82 ± 0.44^a^	10.16 ± 0.57^a^	9.72 ± 0.61^a^
	42	5.63 ± 0.43^c^	6.31 ± 0.45^bc^	7.32 ± 0.41^a^	6.84 ± 0.37^ab^

^a–^^c^Values in the same row without the same uppercase differed significantly (*P* < 0.05).

The gene expressions of IL-2 and INF-γ were examined in the spleen (Table 3). Compared to controls, the expression of spleen IL-2 gene was increased in both M and H groups at day 28 (*P* < 0.05), then returned toward the control level in H group (*P* > 0.05) but not in M group (*P* < 0.05) at day 42. Spleen IFN-γ gene were higher expressed in both M and H groups at day 14 (*P* < 0.05), which was continuously to day 28 (*P* < 0.05), compared to control group.

**Table 3:** Treatment effects on the gene expression abundances of immune factors in the spleen.

Immune factors	Growth point (day)	Treatments			
		Control	L	M	H
IL-2	14	0.40 ± 0.14	0.45 ± 0.12	0.49 ± 0.11	0.46 ± 0.09
	28	0.34 ± 0.08^b^	0.32 ± 0.06^b^	0.62 ± 0.18^a^	0.58 ± 0.07^a^
	42	0.48 ± 0.14^b^	0.39 ± 0.13^b^	0.71 ± 0.07^a^	0.59 ± 0.08^ab^
IFN-γ	14	2.54 ± 0.42^b^	2.64 ± 0.24^b^	3.32 ± 0.67^a^	3.23 ± 0.08^a^
	28	2.08 ± 0.06^b^	2.22 ± 0.52^ab^	3.14 ± 0.13^a^	2.89 ± 0.28^a^
	42	1.08 ± 0.23	1.19 ± 0.17	1.26 ± 0.33	1.16 ± 0.15

^a–^^c^Values in the same row without the same uppercase differed significantly (*P* < 0.05).

### Diversity and structure of cecal microbiota

**Figure 1:**
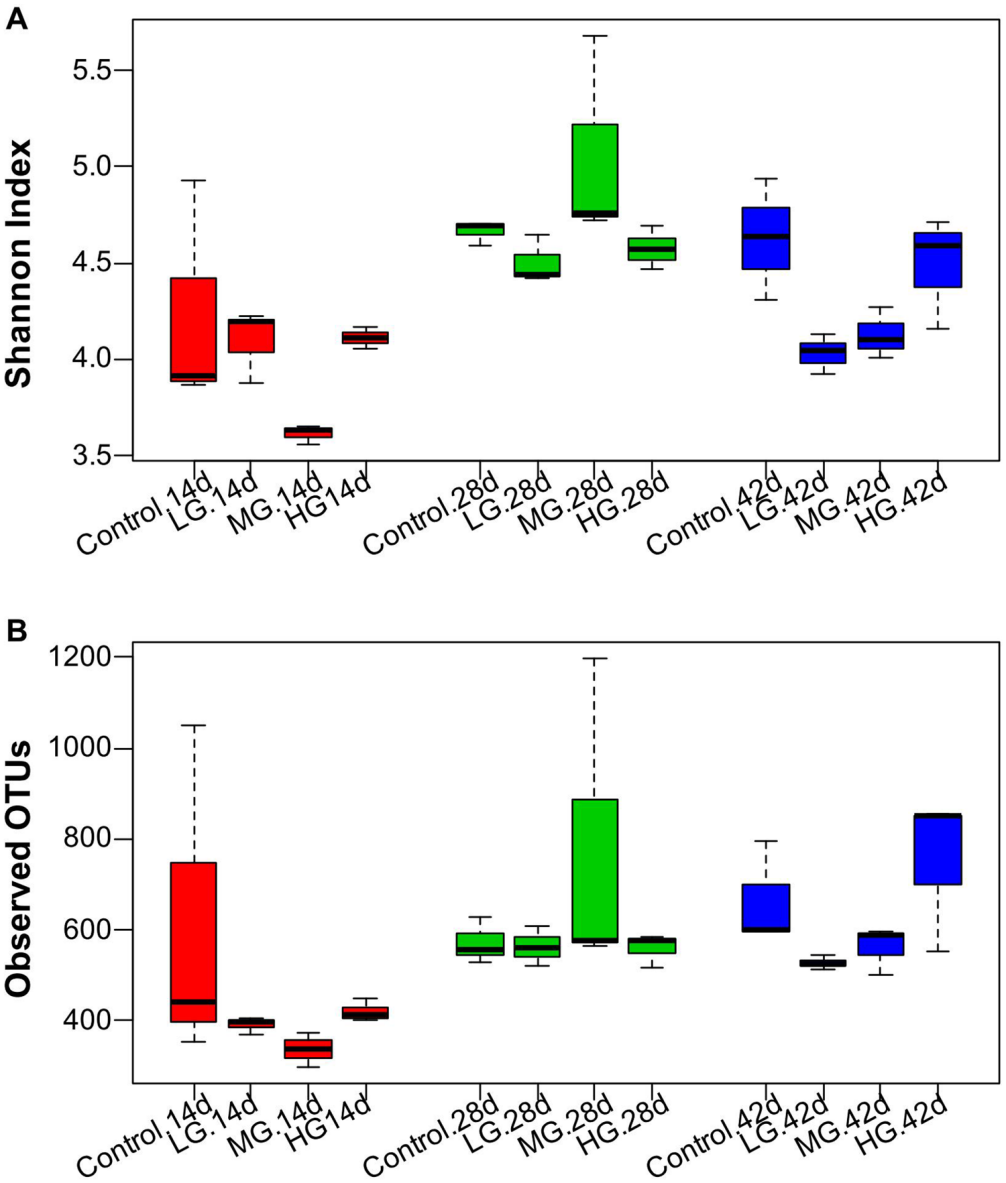
Differences in the gut bacterial community diversity (**A**) and richness (**B**) in chickens fed different levels of bamboo leaf flavone. Alpha diversities were measured by Shannon index and Observed OTUs. The top and bottom boundaries of each box plot indicate the 75th and 25th quartile values, respectively. The horizontal lines within each box represent the median values.

**Figure 2:**
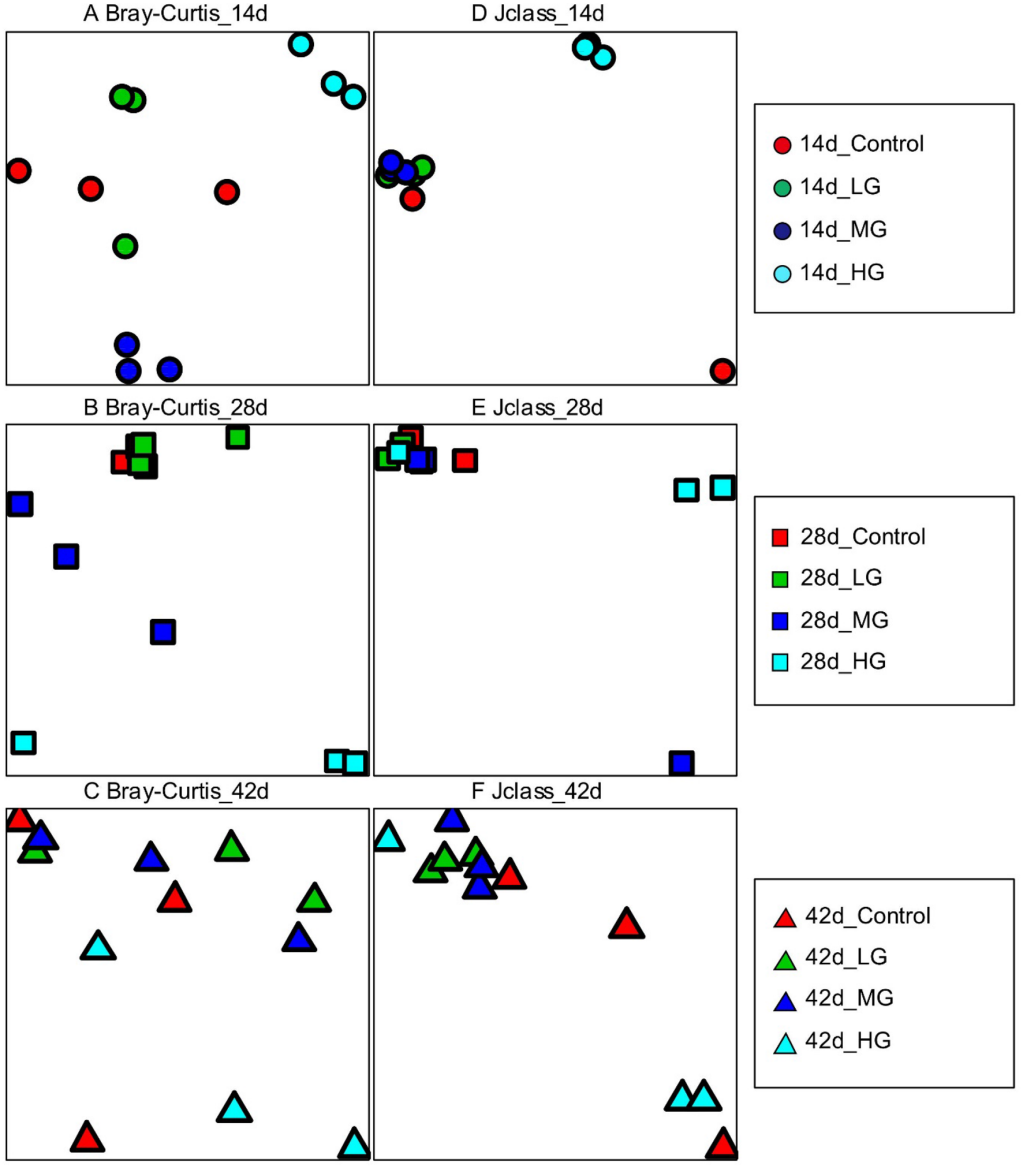
Differences in the gut bacterial community membership and structure in chickens fed different levels of bamboo leaf flavone. Principal Coordinate Analysis (PCoA) of bacterial community structures (**A**) and memberships (**E**) of the microbiota of three age groups with different concentrations of bamboo leaf flavone. PCoA indicated distinct bacterial communities on day 14 (**B**) and (**F**), 28 (**C**) and (**G**), and 42 (**D**). Each symbol represents each sample, red: day 14; green: day 28; and blue: day 42.

The effects of BLF dietary supplement on the composition and diversity of the cecal microbiota of broilers were measured using the next generation sequencing. A total of 1,196,537 high quality reads was generated from 22,374 operational taxonomic units (OTUs), based on > 97% sequencing, which was similar from all 36 samples. Enough sequencing coverage was achieved after attaining a coverage range from 90.10 to 97.95% by normalizing the number of reads to the smallest for each sample (6,919) (Supplementary Table S2). Both the community richness index of Sobs (Observed OTUs) and Chao1 and community diversity index of Shannon and Inverse Simpson are presented in Fig. 1; and it showed there was no treatment effect on the bacterial community diversity and richness during all examined time points (*P* > 0.05, Fig. 1A, B). The dissimilarities between community structure and membership were examined by principal coordinate analysis (PCoA) based on Bray–Curtis and Jclass distances. On the PCoA plots, each symbol represented the bacterial community of a bird (Fig. 2). The community structure of M and H groups on both day 14 and 28 formed distinct clusters compared to control group (Fig. 2A, B), while H group showed clear separation on day 42 (Fig. 2C). The separations were clearly showed in the community membership in all the age groups (Fig. 2D–F). These findings illustrated that the microbial community was influenced by the BLF supplement.

### Relative abundances of the dominant phyla and genera in the cecal microbiota

**Figure 3:**
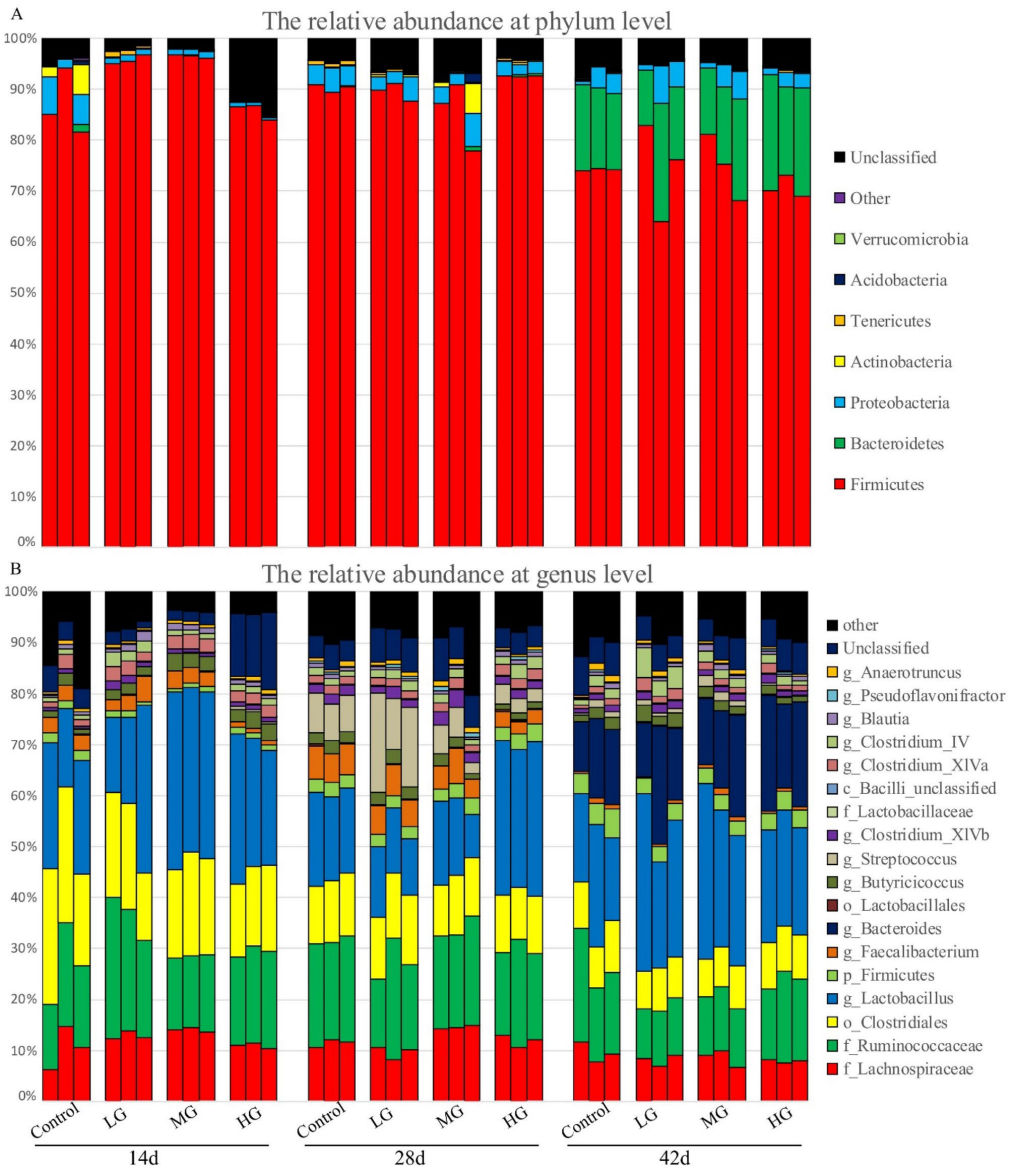
Microbial composition affected by chicken age chicken and the levels of bamboo leaf flavone. The levels of phylum (**A**) and genus (**B**). Each bar represents the relative abundance of each bacterial taxon. The top 5 abundant phylum and 28 abundant genera are listed.

A demonstration was carried out to determine the effect of BFL on bacterial composition and related bacterial community. Therefore, their sequences were binned out into phylotypes according to taxonomic classification of RPD project. After the demonstration by crossed all samples, more than 7 phyla of bacteria were identified, of which *Bacteroidetes*, *Firmicutes*, and *Proteobacteria* were the most dominant ones (Fig. 3A) and 10 unclassified phyla were also found. The greatest change was occurred on post-treatment day 42 with higher levels of *Bacteroidetes* but less *Firmicutes*, compared with those on day 14 and 28. The distribution of the community composition at genus level is illustrated in Fig. 3B. Unclassified *Lachnospiraceae*, *Ruminococcaceae*, *Clostridiales*, and *Lactobacillus, as* the four most abundant genera, were also generated. In addition, the levels of both *Streptococcus* and *Bacteroides* were improved on day 28 and 42, respectively.

**Figure 4:**
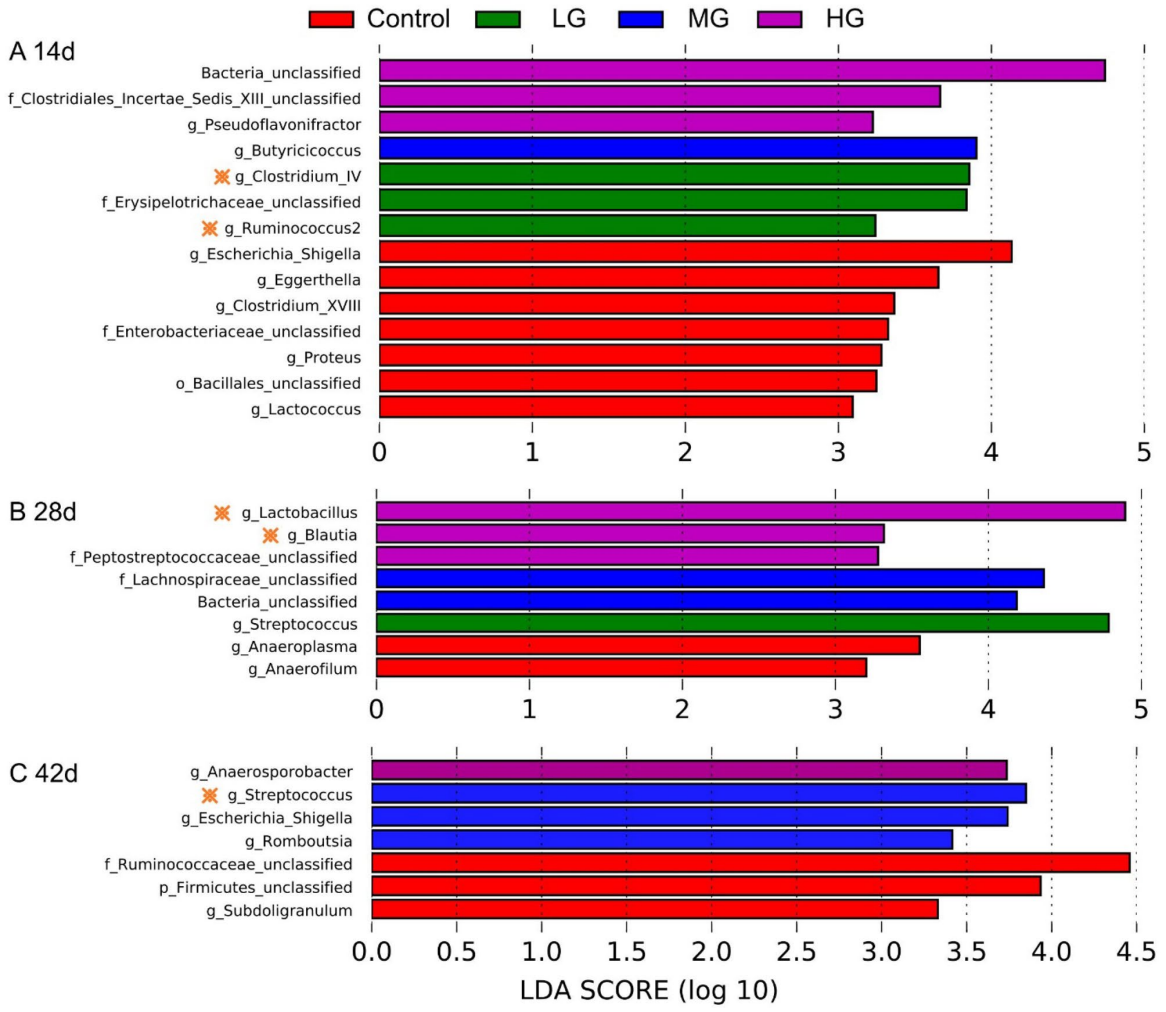
Bacterial taxa affected by chicken age identified by linear discriminant analysis coupled with effect size (LEfSe). The changes of bacterial taxa at day 14 (**A**); day 28 (**B**); and day 42 (**C**).

To further identify specific bacterial taxa associated with the BLF supplement, the linear discriminant analysis effect size (LEfSe) was performed to determine the features of phylotypes based on the logarithmic LDA using the score of 2.0 as the cutoff, with the emphasis on both statistical significance and biological consistency. The results showed several phylotypes were differentially represented in each group among the growth phases (Fig. 4). On day 14 (Fig. 4A), 14 genera (e.g. *Clostridiales_Incertae_Sedis_XIII*, *Clostridium_XVIII*, and *Eggerthella*) were significantly differentially represented among the four groups, while 8 (Fig. 4B) and 7 genera (Fig. 4C) (e.g. *Lactobacillus* and *Anaerosporobacter*) were identified on day 28 and 42, respectively.

### Gut microbiome associated with age and bamboo leaf flavone

The bacterial taxa at the OTUs level associated with age and the BLF supplement were examined by using MaAslin online analysis (https://huttenhower.sph.harvard.edu/galaxy/). A total of 14 bacterial taxa was discovered to be associated with both age and BLF treatment. Especially, one was observed to associate with the BLF dosage (Supplementary Table S2). Among the taxa, the OTUs including Otu147_o_Clostridiales, Otu192_f_Ruminococcaceae, and Otu266_f_Ruminococcaceae were positively coefficient with age and BLF treatment, while Otu002_g_Lactobacillus, Otu012_f_Lachnospiraceae, Otu031_g_Clostridium_XlVa, Otu066_g_Butyricicoccus, Otu168_g_Faecalibacterium, and one OTU belonging to unclassified phylum Firmicutes were either positively or negatively associated with age and BLF treatment. Otu001_g_Lactobacillus and Otu036_f_Ruminococcaceae were negatively associated with both age and the concentration of BLF.

## Discussion

Flavonoids, a kind of natural polyphenolic compounds in plants^16^, have been proven to have a beneficial effect on the immune system of animals^17^. In this study, we confirmed that BLF can improve the immune performance in broilers. The immune organ index has been used as an indicator of the capability of lymphoid cells to produce immune factors in response to stimulations^18^, and our data indicated that BLF has a positive effect on the immune performance of broilers via stimulating the development of the bursa, thymus and spleen. Similar to our findings, previous research has reported that flavonoids modulate immune function in animals. For example, supplementation of alfalfa flavonoids in diets improves growth performance, spleen and bursa weights, and aspartate transaminase activity of Yangzhou geese^19^. In addition, cytokine profile is an important marker of immune status in animals. IFN-γ and IL-2 are two important cytokines in the immune system^19,20^. Both enhance the activity of T and natural killer (NK) cells, playing a major role in the host defense system. The current results further revealed that BLF enhances IFN-γ and IL-2 secretion in bloodstream and *IL-2* and *INF-γ* gene expressions in the spleen to exert immunoregulatory effects. Therefore, the BLF as a feed additive could possess health-promoting properties in chickens.

Numerous studies have reported that chicken intestinal tract harbors complex microbiota which can be altered by various stimuli including dietary factors such as protein levels and feed additives (e.g. probiotics, organic acid, and feed enzymes)^21–23^. Reed et al.^24^ reported that Zn biofortified wheat based diet increased microbial diversity in chickens, particularly increased lactic acid bacteria producing short chain fatty acids. Supplementation of raffinose and stachyose in diets also increased beneficial bacterial populations to promote health in broilers^25^. In addition, the gut microbial ecology plays an important role in the development of the immune response in animals^24,25^. Similarly, we have detected that broilers harbor complex and dynamic bacterial phyla, which dominantly are *Firmicutes*, *Bacteroidetes*, and *Proteobacteria*. Healthy animals show a harmonious interaction between the gastrointestinal tract and resident microbiota to keep biological homeostasis. However, a dysbiosis in their composition and/or function may result in intestinal and extraintestinal diseases^26^. For instance, changes of *Firmicutes* and *Bacteroidetes* contribute to host metabolic disorders through changing energy harvest, lipid metabolism, endocrine function, and inflammatory response^27^.

There is a great interest in the study of small intestinal microbiota as well as the microbiota occupied in the anaerobic cecum in chickens, as the gut microbiota displays a great impact on chicken growth and health^28^. The results from several studies have suggested that plant extracts may modify the composition of the intestinal microbiota to improve the host health^29^. Vidanarachchi et al.^30^ reported that water-soluble carbohydrate extracted from the Cabbage trees (Cordyline australis, Acacia, and Undaria seaweed) increased the number of *Lactobacilli* and reduced harmful bacteria, such as pathogenic coliforms and *C. perfringens*. Li et al.^31^ also reported that flavonoid isoorientin inhibited potential pathogenic bacteria such as *Helicobacter* and *Alistipes* in mice. In addition, it has also suggested that flavonoid extracted from pumelo peel inhibits the growth of pathogens, such as *Escherichia coli* and *Pseudomonas*^32^. Our data showed BLF caused a major difference in bacterial community structure as increased the level of genus *Lactobacillus* and *Ruminococcus* etc. *Lactobacillus* has be used as probiotics with beneficial effects on promoting poultry performance^33,34^ and immunity via modulation of the intestinal microbiome^35,36^. In addition, *Ruminococcus* is commonly found in chicken faeces^37^ with functions in degrading mucin to provide carbon and energy for animals. Taken together, the improved immune functions in BLF fed chickens could be modulated by increasing abundances of beneficial bacterial communities.

The bacterial taxa identified in this study mainly belong to *Clostridiales* and *Lactobacillus* genera; and the families of *Ruminococcaceae* and *Lachnospiraceae* were affected by both age and BLF diet. In addition, the genus of *Faecalibacterium* showed higher abundances on post-treatment day 14 and 28, which is one of the most prominent butyrate producers to provide energy^39^. *Lachnospiraceae,* as a family of anaerobic, spore-forming bacteria, are known to degrade complex polysaccharides to short chain fatty acids^38^. These two bacterial families have been found to be associated with the maintenance of gut health^38^. We observed BLF supplement in chicken also enhanced the presence of *Erysipelotrichaceae* and *Streptococcus*. Lu et al.^40^ demonstrated that the OTUs belonging to *Streptococcus* were more prevalent in the ileum segment than that in the cacum, while Ptak et al.^41^ found that *Streptococcus* abundance was reduced in diets supplemented with Ca, P, and phytase. Generally, findings from the current and previous studies provide a new perspective for understanding the effect of flavones on the chicken gut microbiota and related its funtions. The BLF as a feed additive may inhibit colonization of pathogenic bacteria and increase the abundances of beneficial bacteria to improve the chicken immune response.

Overall, BLF, one of the Chinese herbs, may improve immune function and change the gut microbiota structure in chickens to protect them from colonization of pathogenic bacteria and related inflammatory bowel diseases. The effect of the cecal microbiota on chicken growth performance and production safety has attracted considerable interests since the European Union banned (2006) the use of antimicrobials in livestock production^42^. Particularly, BLF, considered as “prebiotic-like” effects, shapes the gut microbiota to favor other specific gut microbial species that provide healthy benefits to the hosts, such as *Faecalibacterium* and *Lactobacillus* in this study. The current results provided evidence which BLF could be an alternative to antibiotics for improving chicken health and production.

## Methods

### Experimental design and sample collection

The animal experiment was performed at the Poultry Farm of Sichuan Agricultural University. All of the procedures was conducted in accordance with the national standard “Laboratory Animal Requirements of Environment and Housing Facilities” (GB 14925-2001) and approved by the Sichuan Agricultural University’s Institutional Animal Care and Use Committee (Approval number DYY-2018203007).

A total of 300, one-day-old male Arbor Acres broilers (AA) was obtained from Wenjiang Zhengda livestock and poultry Co., Ltd (Sichuan, China), and randomly assigned into 1 of 4 groups in 3 replicates of 25 birds per replicate: A regular diet with BLF supplement at 0 (control), 200 (Low group, L), 400 (Medial group, M), and 800 mg/(kg d) (High group, H). The product of BLF was received from the Zhejiang Saint's Biotechnology Co., Ltd (Zhejiang, China), with the ingredient includes 85% flavonoids of bamboo leaf (mainly contained orientin, homoorientin, vitexin, and isovitexin) and 15% starch (Certificate No: 20130314). Bamboo leaf flavone was extracted by followed the enzyme-assistant extraction method, described previously^43^.

The chickens were vaccinated against the Marek’s disease (at day 1), H9 avian influenza (at day 8), and H5 avian influenza (at day 24), respectively. The corn-soy diet with 21.4% CP and 3,015 kcal of ME/kg was fed from day 1 to day 28, followed by the diet with 19.9% CP and 3,100 kcal of ME/kg from day 29 to day 42. Feed and water were provided ad libitum*.*

The chickens were inspected their activity and healthy status daily. A 5 mL blood was collected from each of 16 sampled birds per group on day 14, 28, and 42, then killed by exsanguination. The spleen, thymus, and bursa from each sampled bird were collected and weighted, respectively. The immune organ index was calculated as organ weight divided by body weight. Blood serum and spleen samples were collected to analyze the concentrations of IL-2 and INF-γ proteins and their gene expressions, respectively. The cecal contents were collected from each replicate from all groups. Considering normalizing the dissimilarity in gut microbiota, three samples per repetition (0.5 g per sample) were mixed. Then, a total of 36 cecal samples was collected in sterilized tubes, and immediately stored at − 80 °C for further microbial DNA extraction.

### DNA extraction and the V4 region of 16S rRNA gene sequencing

Total microbial DNA from each cecal sample was isolated by using the E.Z.N.A.® Stool DNA Kit (Omega, USA) according to the manufacturer’s instructions. The DNA quality was detected by electrophoresis on a 1% agarose gel and then stored at − 20 °C. The bacterial V4 hypervariable region of 16S rRNA gene was amplified by using broadly conserved primer pairs 515F (5′-GTGCCAGCMGCCGCGGTAA-3′) and 806R (5′-GACTACHVGGGTWTCTAAT-3′). The PCR reaction was performed in a total of 25 μL volume including 1 μL (100 mg) DNA template, 0.5 μL each primers, 10 μL Five Prime Hot Master Mix (5 prime: Item No 2200410) and 13 μL PCR Grade H_2_O (MoBio: Item No 17000-11) under the following conditions: an initial denaturation cycle at 94 °C for 3 min, followed by 35 cycles at 94° C for 45 s , 50° C for 60 s annealing, 72° C for 90 s extension, and a final extension at 70° C for 10 min. PCR products were purified by using the QIAGEN Gel Purification Kit (QIAGEN, Dusseldorf, Germany) and quantified by using Qubit. At last, 20 purified amplicons were pooled followed the Qubit value and sequenced on the Illumina MiSeq 2 × 250 platform conducted by Novogene company (Beijing, China). Raw sequences were submitted to NCBI Sequence Read Archive (SRA accession: PRJNA601604).

### Sequence processing and bioinformatics analysis

The quality of Miseq pyrosequencing data were evaluated and analyzed using mothur v1.39.5^44^ followed Miseq SOP (https://www.mothur.org/wiki/MiSeq_SOP) described by Kozich et al.^45^. Briefly, (1) the reads with ambiguous bases and any longer than 275 bp were removed; (2) the chimeric sequences were removed by using UCHIME^46^; (3) the sequences were aligned with the SILVA reference database v128^47^, and the sequences that could not be aligned due to short overlap were removed; and 4) the sequences were pre-clustered to remove sequences that contain possible pyrosequencing errors. At last, the high-quality sequences were assigned to operational taxonomic units (OTUs) based on a sequence identity threshold of 97% and were classified using the RDP classifier^48^. The obtained high-quality data was used for the subsequent analysis.

To reduce the biases caused by sequencing effort in downstream alpha and beta diversity analysis, the reads of each sample were randomly subsampled to 6,918. The OTU-level alpha diversity indices such as Shannon and Sobs (observed OTUs) index were calculated to measure the community diversity and community richness. The beta diversity indices based on Bray–Curtis and Jaccard distance were used to estimate the dissimilarity in community structure and membership, which was applied by the principal coordinate analysis (PCoA) to visualize the dissimilarity of microbial communities.

The relative abundances of the recovered phyla, classes, orders, families, genera, and species were statistically compared among the samples and groups. Specific microbiota species among the different treatment groups were identified using LEfSe (Linear discriminant analysis effect size) (https://huttenhower.sph.harvard.edu/galaxy/^49^, which was performed under the threshold on the logarithmic LDA score > 2.0 for discriminative features. The association between the microbial community (genus) abundance with age and concentration of bamboo leaf flavone was evaluated by MaAsLin (Multivariate Analysis by Linear Models) online.

### Serum IL-2 and INF-γ concentration analysis

The blood sample was kept at room temperature for 1 h and then centrifuged at 4,550×g at 4 °C for 5 min. Supernatant liquid was collected and stored at −20 °C for further analysis. The concentrations of IL-2 and INF-γ were determined by ELISA (Nanjing Jiancheng Biological Reagent co., LTD, China) followed the company’s instruction.

### RNA isolation and quantitative real-time polymerase chain reaction

Total RNA from the spleen was extracted by using Trizol reagent (TIANGEN, Beijing, China) according to the manufacturer’s protocol. The RNA quality was examined by electrophoresis on a 1% agarose gel. One microgram of total RNA from each sample was reverse transcribed into cDNA using the PrimeScript RT reagent kit with cDNA eraser (TaKaRa, Dalian, China). The following PCR reaction program was applied: 3 min heating at 95 °C, followed by 40 cycles of denaturation (10 s at 95 °C), annealing (20 s at 57 °C), and extension (15 s at 72 °C). Primer pairs used for the reverse transcription were listed in Supplementary Table S1. Relative mRNA expression levels of each target gene were calculated using the 2^−∆∆Ct^ method.

### Statistical analysis

The effects of BLF were analyzed using one-way ANOVA. The major factors were with or without BLF and BLF concentrations. The differences of alpha diversity indices were analyzed by using Kruskal Wallis test among treatment groups. The results were presented as: mean + SEM. Statistical significance was considered at *P* < 0.05.

## Supplementary information

Supplementary information
