# Supplementary material for: Bamboo leaf flavone changed the community of cecum microbiota and improved the immune function in broilers
Source: Sci Rep. 2020 Jul 23;10:12324. doi: 10.1038/s41598-020-69010-1 (PMC7378082; doi:10.1038/s41598-020-69010-1)
Supplement: Supplementary file 1 — Supplementary information [file 41598_2020_69010_MOESM1_ESM.docx]

Bamboo Leaf Flavone Changed the Community of Cecum Microbiota and Improved the Immune Function in Broilers

Gang Shu ^1 #^, Fanli Kong ^2 #^, Dan Xu ^1 #^, Lizi Yin ^1^, Changliang He ^1^, Juchun Lin ^1^, Hualin Fu ^1^, Kaiyu Wang ^1^, Yaofu Tian ^3^, Xiaoling Zhao ^3 *^

**Supplementary Table S1 Primer information for qRT-PCR assays**

| Gene Name | 5’-3’ | GenBank Accession No. |
| --- | --- | --- |
| β-actin | F-TGTGCTGTCCCTGTATGCCTC | X00182 |
|  | R-GAGGCATACAGGGACAGCACA |  |
| IL-2 | F-CTTTGGCTGTATTTCGGTAGCA | AF000631 |
|  | R-CACTCCTGGGTCTCAGTTGGTG |  |
| IFN-γ | F-GACAAGTCAAAGCCGCACA | X99774 |
|  | R-TCAAGTCGTTCATCGGGAGC |  |

| **Supplementary Table S2 Sample information** | | | | | | | | |
| --- | --- | --- | --- | --- | --- | --- | --- | --- |
| Group | Treatment (mg/(kg*d)) | Age | SeqNo. | Coverage | Shannon | Invsimpson | Sobs | Chao |
| aRS1.1 | 0 | 14d | 23646 | 0.961266 | 3.86477 | 19.665408 | 442 | 1203.234 |
| aRS1.2 | 0 | 14d | 26000 | 0.972684 | 3.916293 | 20.25824 | 353 | 875.5294 |
| aRS1.3 | 0 | 14d | 83756 | 0.905911 | 4.924633 | 32.765745 | 1049 | 2651.841 |
| aRS2.1 | 200 | 14d | 7806 | 0.968059 | 4.192532 | 28.091023 | 398 | 1021.333 |
| aRS2.2 | 200 | 14d | 19696 | 0.967625 | 4.224678 | 28.056379 | 406 | 1211.677 |
| aRS2.3 | 200 | 14d | 28209 | 0.970805 | 3.876125 | 15.945551 | 369 | 1120.889 |
| aRS3.1 | 400 | 14d | 39590 | 0.979477 | 3.555288 | 13.013545 | 298 | 592.4412 |
| aRS3.2 | 400 | 14d | 26365 | 0.970227 | 3.637298 | 13.729616 | 374 | 944.6757 |
| aRS3.3 | 400 | 14d | 18422 | 0.974996 | 3.652785 | 14.342917 | 338 | 775.5882 |
| aRS4.1 | 800 | 14d | 24906 | 0.967192 | 4.054251 | 17.800417 | 448 | 982.3958 |
| aRS4.2 | 800 | 14d | 66401 | 0.972539 | 4.172288 | 22.282034 | 413 | 821.0682 |
| aRS4.3 | 800 | 14d | 68419 | 0.974418 | 4.10832 | 21.367484 | 400 | 731.4043 |
| bRS1.1 | 0 | 28d | 45955 | 0.96459 | 4.591504 | 34.401115 | 529 | 1151.708 |
| bRS1.2 | 0 | 28d | 8663 | 0.961122 | 4.697009 | 40.207686 | 557 | 1263.784 |
| bRS1.3 | 0 | 28d | 34926 | 0.950715 | 4.703439 | 38.678682 | 626 | 1661.179 |
| bRS2.1 | 200 | 28d | 43099 | 0.951438 | 4.418305 | 19.721412 | 610 | 1517.742 |
| bRS2.2 | 200 | 28d | 40653 | 0.959965 | 4.646869 | 37.760781 | 561 | 1256.018 |
| bRS2.3 | 200 | 28d | 17669 | 0.964012 | 4.443799 | 25.428014 | 522 | 1073.357 |
| bRS3.1 | 400 | 28d | 17867 | 0.961122 | 4.763839 | 46.906866 | 564 | 1145.387 |
| bRS3.2 | 400 | 28d | 6919 | 0.957508 | 4.721256 | 43.871654 | 578 | 1475.313 |
| bRS3.3 | 400 | 28d | 76636 | 0.900997 | 5.677354 | 98.396248 | 1199 | 2740.25 |
| bRS4.1 | 800 | 28d | 52455 | 0.965457 | 4.464112 | 28.011326 | 517 | 1034.109 |
| bRS4.2 | 800 | 28d | 30911 | 0.962567 | 4.692877 | 32.630385 | 583 | 1022.618 |
| bRS4.3 | 800 | 28d | 27248 | 0.9617 | 4.570897 | 25.426933 | 578 | 1092.412 |
| cRS1.1 | 0 | 42d | 47333 | 0.94479 | 4.9383 | 35.81085 | 796 | 1489.057 |
| cRS1.2 | 0 | 42d | 7891 | 0.956352 | 4.30673 | 18.93319 | 595 | 1156.123 |
| cRS1.3 | 0 | 42d | 12372 | 0.960977 | 4.637222 | 28.230157 | 600 | 1065.577 |
| cRS2.1 | 200 | 42d | 24348 | 0.958809 | 4.041758 | 15.599067 | 543 | 1138.147 |
| cRS2.2 | 200 | 42d | 47522 | 0.9617 | 3.92315 | 12.673886 | 513 | 1116.103 |
| cRS2.3 | 200 | 42d | 27529 | 0.96011 | 4.132649 | 18.817833 | 525 | 1254.808 |
| cRS3.1 | 400 | 42d | 17887 | 0.95245 | 4.102069 | 13.393539 | 596 | 1633.615 |
| cRS3.2 | 400 | 42d | 20104 | 0.955774 | 4.272263 | 17.30339 | 587 | 1253.643 |
| cRS3.3 | 400 | 42d | 16277 | 0.967481 | 4.009056 | 12.660959 | 501 | 901 |
| cRS4.1 | 800 | 42d | 45572 | 0.959965 | 4.158543 | 13.081161 | 551 | 1300.529 |
| cRS4.2 | 800 | 42d | 45422 | 0.936985 | 4.711113 | 19.890694 | 854 | 1624.976 |
| cRS4.3 | 800 | 42d | 48063 | 0.93554 | 4.593272 | 16.493939 | 851 | 1769.843 |

**Supplementary Table S3 Maslin's data for bacteria taxa**

|  | OTUs | Value | Coefficient | P.value |
| --- | --- | --- | --- | --- |
| 1 | Otu001_g_Lactobacillus | TimePoint28d | -0.1328243 | 2.3367E-07 |
|  |  | TimePoint42d | -0.1643638 | 2.9417E-09 |
| 2 | Otu002_g_Lactobacillus | TimePoint28d | -0.0718028 | 0.0004319 |
|  |  | TimePoint42d | 0.18914209 | 1.7457E-11 |
|  |  | treatment800mg/g | 0.06930703 | 0.00245933 |
| 3 | Otu012_f_Lachnospiraceae_unclassified | TimePoint42d | -0.034724 | 2.6674E-06 |
|  |  | treatment400mg/g | 0.02082331 | 0.00544727 |
| 4 | Otu030_f_Lachnospiraceae_unclassified | TimePoint28d | -0.0395583 | 7.3828E-06 |
|  |  | TimePoint42d | -0.0575946 | 6.6213E-09 |
| 5 | Otu031_g_Clostridium_XlVa | TimePoint28d | 0.01505126 | 0.00270108 |
|  |  | treatment200mg/g | 0.01783251 | 0.00216281 |
| 6 | Otu036_f_Ruminococcaceae_unclassified | TimePoint28d | -0.0347339 | 6.1823E-05 |
|  |  | TimePoint42d | -0.0535401 | 5.5546E-08 |
|  |  | treatment200mg/g | -0.0254287 | 0.00610119 |
|  |  | treatment400mg/g | -0.0271649 | 0.00366359 |
| 7 | Otu066_g_Butyricicoccus | TimePoint28d | 0.0348485 | 6.3037E-07 |
|  |  | treatment200mg/g | -0.0205187 | 0.00319867 |
| 8 | Otu147_o_Clostridiales_unclassified | TimePoint28d | 0.03256159 | 1.4013E-11 |
|  |  | TimePoint42d | 0.02472477 | 6.521E-09 |
|  |  | treatment800mg/g | 0.0105817 | 0.00596757 |
| 9 | Otu168_g_Faecalibacterium | TimePoint42d | -0.0164151 | 0.00014228 |
|  |  | treatment200mg/g | 0.01770962 | 0.00031477 |
| 10 | Otu190_unclassified | TimePoint28d | 0.0153157 | 9.1968E-06 |
|  |  | TimePoint42d | 0.01810776 | 6.0366E-07 |
|  |  | treatment800mg/g | 0.00991578 | 0.00555556 |
| 11 | Otu192_f_Ruminococcaceae_unclassified | TimePoint28d | 0.00788981 | 0.00281467 |
|  |  | treatment200mg/g | 0.01126698 | 0.00035684 |
| 12 | Otu194_unclassified | TimePoint28d | 0.02100356 | 1.2832E-08 |
|  |  | TimePoint42d | 0.0216803 | 6.704E-09 |
|  |  | treatment200mg/g | 0.01188106 | 0.00068817 |
|  |  | treatment800mg/g | 0.00973516 | 0.00417548 |
| 13 | Otu195_p_Firmicutes_unclassified | TimePoint42d | -0.0128835 | 0.00026702 |
|  |  | treatment400mg/g | 0.01363367 | 0.00068756 |
| 14 | Otu199_unclassified | TimePoint42d | 0.00668375 | 0.00523523 |
|  |  | treatment400mg/g | 0.00866411 | 0.00202283 |
| 15 | Otu266_f_Ruminococcaceae_unclassified | TimePoint28d | 0.00682117 | 0.00013051 |
|  |  | TimePoint42d | 0.01096676 | 7.6738E-08 |
|  |  | treatment800mg/g | 0.00991221 | 5.3496E-06 |
